# Prevalence and Prognostic Significance of Preoperative Anemia in Radical Cystectomy Patients: A Multicenter Retrospective Observational Study

**DOI:** 10.1016/j.euros.2025.05.002

**Published:** 2025-06-04

**Authors:** Ernest Kaufmann, Luca Antonelli, Luca Afferi, Vincenzo Asero, Francesco Prata, Silvia Rebuffo, Alessandro Veccia, Meftun Culpan, Karl Tully, Luis Ribeiro, Thierry Roumeguere, Kees Hendricksen, Luca Lambertini, Renate Pichler, Nicola Pavan, Jeremy Yuen-Chun Teoh, Mathieu Roumiguié, Gerald Bastian Schulz, Francesco Soria, Aamer AlGhamlas, Pierre-Emmanuel Desprez, Luca Orecchia, Cedric Poyet, Majed Alrumayyan, Michael Rink, Stefania Zamboni, Maria Riaza Montes, Steven Okoye, Mattia Lo Re, Wojciech Krajewski, Luke Lavallee, Marian Severin Wettstein, Marco Moschini, Christian Daniel Fankhauser

**Affiliations:** a Department of Urology, Luzerner Kantonsspital, University of Lucerne, Lucerne, Switzerland; b Department of Urology, Sapienza University of Rome, Rome, Italy; c Department of Urology, Fondazione Policlinico Universitario Campus BIo-Medico of Rome, Rome, Italy; d IRCCS Ospedale Policlinico S. Martino, Genoa, Italy; e Department of Surgical and Diagnostic Integrated Sciences, University of Genoa, Genoa, Italy; f Department of Urology, Azienda Ospedaliera Universitaria Integrata Verona, Verona, Italy; g Department of Urology, Faculty of Medicine, Istanbul Medeniyet University, Istanbul, Turkey; h Department of Urology and Neurourology, Marien Hospital Herne, Ruhr University Bochum, Bochum, Germany; i St. George’s University Hospital, London, UK; j Service d’Urologie, Hôpital Universitaire de Bruxelles, Université Libre de Bruxelles, Bruxelles, Belgium; k Department of urology, Netherlands Cancer Institute, Amsterdam, The Netherlands; l Unit of Oncologic Minimally Invasive Urology and Andrology - Careggi Hospital, Department of Clinical and Experimental Medicine, University of Florence, Florence, Italy; m Department of Urology, Medical University of Innsbruck, Innsbruck, Austria; n Urology Clinic, Department of Precision Medicine in Medical, Surgical and Critical Care, University of Palermo, Palermo, Italy; o S.H. Ho Urology Centre, Department of Surgery, The Chinese University of Hong Kong, Hong Kong, China; p Li Ka Shing Institute of Health Sciences, The Chinese University of Hong Kong, Hong Kong, China; q Department of Urology, Medical University of Vienna, Vienna, Austria; r Department of Urology, CHU-IUCT, Toulouse, France; s Department of Urology, LMU Munich, Munich, Germany; t Division of Urology, Department of Surgical Sciences, AOU città della Salute e della Scienza di Torino, Torino School of Medicine, Torino, Italy; u Urology, GRC 5 Predictive Onco-Uro, AP-HP, Pitie-Salpetriere Hospital, Sorbonne University, Paris, France; v Urology Department, Claude Huriez Hospital, CHU Lille, Lille, France; w Urology Unit, Department of Surgical Sciences, Tor Vergata University Hospital, University of Rome Tor Vergata, Rome, Italy; x Department of Urology, Stadtspital Zürich Triemli, Zürich, Switzerland; y Division of Urology, Department of Surgery, Princess Margaret Cancer Centre, University Health Network, Toronto, Ontario, Canada; z Department of Urology, Marienkrankenhaus Hamburg, Hamburg, Germany; aa Unit of Urology, Department of Medical and Surgical Specialities, Radiological Science and Public Health, ASST Spedali Civili di Brescia, University of Brescia, Brescia, Italy; bb Galdakao-Usansolo Hospital, Vizcaya, Spain; cc Department of Urology, Charité-Universitätsmedizin Berlin, Berlin, Germany; dd Unit of Urological Robotic Surgery and Renal Transplantation, University of Florence, Careggi Hospital, Florence, Italy; ee Department of Minimally Invasive and Robotic Urology, Wrocław Medical University, Wrocław, Poland; ff Division of Urology, Department of Surgery, University of Ottawa and Ottawa Hospital Research Institute, Ottawa, ON, Canada; gg Department of Uro-Oncology, University of Toronto, Toronto, ON, Canada; hh Department of Urology and Division of Experimental Oncology, Urological Research Institute, Vita-Salute San Raffaele, Milan, Italy

**Keywords:** Anemia, Bladder cancer, Hemoglobin, Patient blood management, Radical cystectomy, Survival outcomes

## Abstract

**Background and objective:**

Preoperative anemia is common in patients undergoing radical cystectomy for bladder cancer, but its prevalence and impact on outcomes remain poorly characterized across different health care settings. This study aims to assess the prevalence of preoperative anemia, evaluate its current management practices, and determine its association with postoperative and oncological outcomes in patients undergoing radical cystectomy.

**Methods:**

We retrospectively analyzed 4886 patients with nonmetastatic bladder cancer who underwent radical cystectomy across 28 centers in 13 countries. Multivariable regression models identified the predictors of preoperative hemoglobin levels and postoperative blood transfusions. Survival outcomes were assessed using Kaplan-Meier and Cox proportional hazards regression analyses.

**Key findings and limitations:**

Preoperative anemia was present in 44% of women and 48% of men. Among anemic patients, 73% received no blood management interventions. Higher hemoglobin levels before transurethral resection of a bladder tumor correlated with higher levels before cystectomy and fewer postoperative blood transfusions (odds ratio: 0.98, 95% confidence interval [CI]: 0.97–0.99, *p* < 0.001). Higher preoperative hemoglobin levels were associated with lower 90-d mortality rates (hazard ratio: 0.98, 95% CI: 0.97–0.99, *p* < 0.001) and independently predicted reduced all-cause mortality, cancer-specific mortality, and disease relapse.

**Conclusions and clinical implications:**

Preoperative anemia is prevalent and undertreated in patients undergoing radical cystectomy, and is independently associated with adverse perioperative and oncological outcomes. This highlights the need for further research regarding the potential benefits of implementing systematic preoperative anemia management.

**Patient summary:**

This study found that low blood counts before bladder removal surgery are common, often untreated, and linked to worse outcomes. Early treatment of low blood counts before surgery could improve results for patients.

## Take Home Message

Anemia prior to radical cystectomy is common affecting almost 50% of patients, whereas treatment is very rare. Preoperative anemia is significantly associated with worse postoperative outcomes; therefore, the impact of early correction of anemia after transurethral resection of a bladder tumor on perioperative and long-term oncological outcomes needs to be evaluated in further prospective studies.

## 1 Introduction

Anemia affects approximately 50% of oncological patients and may arise due to the malignancy itself (eg, bleeding from the tumor) or the treatment, including postoperative bleeding after surgical interventions, such as transurethral resection of the bladder tumor (TURBT) or radical cystectomy, chemotherapy, radiation therapy, nutritional deficiencies, hemolysis, endocrine disorders, or chronic inflammatory diseases [1]. Single-institution studies have reported anemia in 10–50% of patients scheduled for radical cystectomy, suggesting associations between anemia or blood transfusions and worse recurrence-free, cancer-specific, and overall survival [2,3]. However, these findings are limited by their single-center nature and sample sizes, restricting adjustment for significant confounders [2,4].

Preoperative anemia and blood transfusions are linked to increased postoperative complications affecting gastrointestinal, genitourinary, pulmonary, and renal systems, as well as higher infection rates and health care costs [3,[5], [6], [7], [8]]. Given the worldwide shortage of blood products, minimization of transfusions through preventative anemia optimization is crucial. Therefore, studying patient blood management strategies to improve hemoglobin (Hb) levels using folate, vitamin B12, iron, or erythropoietin (EPO) supplementation before cystectomy is imperative.

We hypothesize that in a large patient cohort, preoperative anemia will remain to have a significant negative impact on perioperative outcomes after adjusting for possible confounders. To address the abovementioned critical research gaps, our study undertakes a large-scale, multinational, multicenter investigation with the following three objectives: first, to determine the prevalence of preoperative anemia in cystectomy patients and assess the frequency of Hb optimization interventions; second, to identify risk factors associated with anemia before cystectomy and blood transfusions; and third, to evaluate the relationship between preoperative anemia and both perioperative and long-term survival outcomes, while adjusting for significant confounding factors. This comprehensive approach aims to provide robust insights into anemia management in radical cystectomy across diverse health care settings.

## 2 Methods

### 2.1 Study design and setting

This retrospective observational cohort study utilized the data of 28 institutions in 13 countries across Europe, North America, and Asia. The Declaration of Helsinki, the Guidelines on Good Clinical Practice issued by the European Medicines Agency, Swiss law and regulatory authority requirements, and the Strengthening the Reporting of Observational Studies in Epidemiology (STROBE) were followed for this study.

### 2.2 Patients

We included patients diagnosed with localized or locally advanced bladder cancer who underwent radical cystectomy at the participating centers between 1990 and 2021. Patients with metastatic bladder cancer or incomplete data on Hb levels before cystectomy were excluded. Each participant or his/her guardian provided informed consent before participation, or consent was waived by the local ethical committee. This study received approval from the Swiss Ethical Committee (ID 2022-00538) and local institutional review boards of each institution, and all data were anonymized to protect the patients’ identities.

### 2.3 Variables and data sources

We retrospectively collected data in a standardized dataset for bladder cancer patients from medical records. Data were collected at various time points, including bladder cancer diagnosis; initiation of neoadjuvant chemotherapy, radical cystectomy, and adjuvant therapy; and long-term follow-up.

The collected data included patient characteristics; baseline variables such as age, gender, comorbidities, and performance status; histological and cancer staging information obtained during TURBT; laboratory values; neoadjuvant treatment regimens; characteristics of the radical cystectomy; adjuvant and systemic treatment regimens; survival outcomes; and thromboembolic and bleeding events. The registry structure was managed centrally, with data being aggregated, normalized, and stored securely.

Anemia thresholds were set, as defined by the World Health Organization (WHO), as Hb <130 g/l in male adults and <120 g/l in female adults [9]. Further clinical definitions are described in the Supplementary material.

### 2.4 Study size

The size of the study cohort was determined based on the availability of comprehensive data from the participating institutions over the study period.

### 2.5 Exposure

The primary exposure in this study was preoperative Hb levels measured before radical cystectomy. Hb levels were routinely assessed twice: once before TURBT and once before cystectomy. These measurements aimed to investigate the potential influence of preoperative Hb levels on patient outcomes after surgery. The data on Hb levels were collected alongside other laboratory values and risk factors for bleeding and venous thromboembolism, ensuring a comprehensive evaluation of each patient’s preoperative status.

### 2.6 Outcomes

The primary outcomes of the study comprised anemia before cystectomy, disease relapse (DR), cancer-specific mortality (CSM), all-cause mortality (ACM), and 90-d mortality. Outcomes were defined as follows: DR: measured from the day of cystectomy until the progression of the disease, as detected by imaging studies; CSM and ACM: determined from the date of cystectomy until death attributed to bladder cancer and any cause, respectively; and 90-d mortality: death attributed to bladder cancer or any cause within the first 90 d after cystectomy. Patients were followed up through medical chart reviews or death certificates, with censoring (coded as 0) at the date of the last follow-up and presenting results to a maximum of 5 yr of follow-up. This decision was based on the fact that most patients had follow-up data limited to ≤5 yr, with only a small subset of patients having follow-up beyond this timeframe. Furthermore, current and historical urological guidelines generally recommend structured follow-up primarily within the first 5 yr after treatment, as the risk of recurrence and disease progression decreases significantly thereafter.

### 2.7 Assumed confounding

Our analysis of the influence of preoperative Hb levels on oncological outcomes considers potential confounders that might distort the results. The predictors included in the models (including age, smoking status, Eastern Cooperative Oncology Group [ECOG] performance status, pathological staging [pN+ and pT stage], and chemotherapy use) were selected based on a review of the literature, as these factors have previously been shown to influence oncological and perioperative outcomes in bladder cancer patients. A detailed summary of these predictors and their supporting evidence is provided in the Supplementary material. To control for confounding, these factors were included as covariates in our multivariable models.

### 2.8 Bias

The efforts to minimize a bias included standardization of data collection, and exclusion of patients with missing baseline Hb data or those with metastatic disease at diagnosis.

### 2.9 Statistical analysis

A Shapiro-Wilk test was used to check for normal distribution of the demographic data for continuous variables. A multivariable linear regression model was run to identify the predictors of Hb levels before cystectomy (as a continuous variable) and logistic regression to identify the predictors of postoperative blood transfusions (as a binary variable) during the treatment course. A survival analysis using the Kaplan-Meyer method was utilized for an unadjusted analysis of the influence of preoperative Hb levels on survival and relapse. Significance was assessed with a log-rank test. To examine the association between preoperative Hb levels and oncological outcomes, multivariable Cox proportional hazards regression was performed. To examine the association between preoperative Hb levels and 90-d mortality, Cox logistic regression was performed. For this purpose, it was ensured before the analysis that all patients were followed up for at least 90 d. Statistical significance for all analyses was defined at *p* < 0.05. Statistical analyses were conducted using the R programming language (R Core Team, 2022).

## 3 Results

Between January 1990 and December 2021, our multicenter study encompassed 4886 patients across 28 centers in 13 countries. We excluded 441 patients due to the absence of precystectomy Hb values. Consequently, our final cohort comprised 4445 patients with a mean age of 69 yr, of whom 1007 (23%) were female. The median follow-up of the surviving patients was 25 mo (interquartile range [IQR] 11–53 mo). The baseline characteristics are shown in Table 1. The median Hb levels were recorded to be 134 g/l (IQR 120–146 g/l) prior to TURBT and 128 g/l (IQR 111–142 g/l) prior to radical cystectomy.

**Table 1:** Baseline characteristics of the study population

Variables	*N* = 4445 (100)
Gender	
Male	3436 (77.3)
Missing	1 (0)
Age	70 (62–77)
Medical history	
Hypertension	2410 (54.2)
Renal disease (dialysis, transplant, creatinine >2.26 mg/dl or >200 µmol/l)	247 (5.6)
Missing	595 (13.4)
Liver disease (cirrhosis, bilirubin >2× normal with AST/ALT/AP >3× normal)	63 (1.4)
Missing	594 (13.4)
History of stroke	237 (5.3)
Missing	595 (13.4)
Diabetes	796 (17.9)
Missing	20 (0)
Alcohol abuse (>8 drinks/wk)	199 (4.5)
Missing	1019 (22.9)
Smoking history	
Nonsmoker	1563 (35.2)
Current smoker	1361 (30.6)
Ex-smoker	1520 (34.2)
Missing	178 (4.0)
T stage at TURBT	
pTa	32 (0.7)
pT1	1234 (27.8)
pT2	2648 (59.6)
pTis	350 (7.9)
Missing	180 (4.0)
N stage at TURBT	
cN0	3985 (89.7)
cN+	427 (9.6)
Missing	700 (15.7)
1973 WHO grade	
G1	65 (1.5)
G2	223 (5)
G3	2996 (67.4)
Missing	1160 (26.1)
2004 WHO grade	
Low grade	643 (14.5)
High grade	3801 (85.5)
Missing	494 (11.1)
Patients receiving neoadjuvant treatment	824 (18.5)
Missing	17 (0)
Patients receiving adjuvant treatment	677 (15.2)
Missing	188 (4.2)
Type of surgery	
Open	3934 (88.5)
Laparoscopic	81 (1.8)
Robot assisted	424 (9.5)
Missing	5 (0)
T-stage cystectomy specimen	
pT0, pTa, pTis	956 (21.7)
pT1	569 (12.9)
pT2	960 (21.8)
pT3	1294 (29.4)
pT4	620 (14.1)
Missing	45 (1)

ALT = alanine aminotransferase; AP = alkaline phosphatase; AST = aspartate aminotransferase; G = grade; TURBT = transurethral resection of a bladder tumor; WHO = World Health Organization.

Medians and interquartile ranges for continuous variables, and counts and percentages for ordinal variables.

Anemia before TURBT was observed in 1008 (38%) males and 445 (33%) females, based on the WHO thresholds of 130 and 120 g/l, respectively (Table 2). At the time of cystectomy, anemia was observed in 1674 (49%) males and 445 (44%) females. Among those patients with an Hb level of <130 g/l, 1031 patients (73%) received no specific Hb optimization. A total of 324 patients (23%) received packed red blood cells, 4% oral or intravenous iron, 3% vitamin B12, 4% folate, and 1.5% EPO injections. Postcystectomy low anemia/bleeding events necessitating medical intervention (transfusion of packed red blood cells, or radiological, endoscopic, or surgical intervention) were 10%.

**Table 2:** Anemia before cystectomy and treatment of anemia

Hemoglobin level before TURBT/at diagnosis		
Female	128 (114–140)	
Male	136 (121–147)	
Hemoglobin level before cystectomy		
Female	123 (108–135)	
Male	130 (113–144)	
Anemia before TURBT (male <130 g/l, female <120 g/l)		
Female	445 (33)	
Male	1008 (38)	
Anemia before cystectomy		
Female	445 (44)	
Male	1674 (49)	
Therapy between TURBT and cystectomy (Hb before TURBT)	Hb >130 g/l (*n* = 2031)	Hb <130 g/l (*n* = 1419)
Transfusion of packed red cells	142 (7)	324 (22.8)
EPO injections	16 (0.8)	22 (1.6)
Folate supplementation	9 (0.4)	52 (3.7)
Iron supplementation	11 (0.5)	51 (3.6)
Vitamin B12 therapy	12 (0.6)	47 (3.3)
No therapy	1855 (91.3)	1031 (72.7)
Bleeding events, requiring medical intervention		
During neoadjuvant treatment	14 (1.7)	
After cystectomy	429 (9.7)	
During adjuvant treatment	13 (1.9)	

EPO = erythropoietin; Hb = hemoglobin; TURBT = transurethral resection of a bladder tumor.

Medians and interquartile ranges for continuous variables, and counts and percentages for ordinal variables.

A multivariable linear regression analysis highlighted several factors influencing precystectomy Hb levels (Table 3). Higher Hb levels at TURBT and a lower Charlson comorbidity score were associated with higher Hb levels before cystectomy (*p* < 0.001). In contrast, increasing age, administration of neoadjuvant chemotherapy, and higher tumor stage at TURBT ≥pT2 were associated with lower Hb levels before cystectomy (*p* < 0.001). EPO injections (*p* = 0.025) and vitamin B12 supplementation (*p* = 0.026) were also associated with lower Hb values before cystectomy. No statistical association could be confirmed for American Society of Anesthesiologists scores or the use of iron or folate supplementation.

**Table 3:** Multivariable linear regression for predictors of hemoglobin before cystectomy

Variable	Estimate	95% CI	*p* value
*Univariable regression*			
Hb value before TURBT (g/l; continuous)	0.64	0.61–0.67	**<0.001**
*Multivariable regression*			
Age (continuous)	–0.12	–0.18 to –0.06	**<0.001**
ASA score (reference = 1)			
2	1.63	–0.42 to 3.67	0.12
3	1.27	–0.74 to 3.28	0.21
4	2.56	–1.96 to 7.08	0.27
Charlson comorbidity score (reference = 0)			
1	–2.01	–4.25 to 0.23	0.08
2	–1.13	–2.94 to 0.68	0.22
3	–1.50	–3.56 to 0.57	0.16
4	–2.49	–4.82 to –0.17	**0.036**
5	–4.68	–7.41 to –1.95	**<0.001**
6	–5.19	–7.98 to –2.41	**<0.001**
Clinical nodal stage cN+ (reference = cN0)	0.59	–1.20 to 2.38	0.518
Hb value before TURBT (g/l; continuous)	0.64	0.60 to 0.67	**<0.001**
Packed red blood cells after TURBT (reference = no therapy)	–1.48	–3.66 to 0.70	0.18
EPO injection (reference = no EPO)	–7.66	–14.37 to –0.96	**0.025**
Iron supplementation (reference = no iron)	–1.10	–5.75 to 3.56	0.65
Vitamin B12 therapy (reference = no vitamin B12)	–6.90	–13.00 to –0.81	**0.026**
Folate therapy (reference = no therapy)	2.96	–3.17 to 9.09	0.34
Alcohol abuse >8 drinks/wk (reference = no abuse)	–1.64	–4.16 to 0.87	0.20
Diabetes (reference = no diabetes)	–0.27	–1.76 to 1.23	0.73
Smoking history (reference = nonsmoker)	0.56	–0.77 to 1.90	0.41
Neoadjuvant therapy (reference = no therapy)	–9.57	–11.07 to –8.06	**<0.001**
Tumor stage ≥pT2 (reference = <pT2)	–2.20	–3.41 to –0.99	**<0.001**

ASA = American Society of Anesthesiologists; CI = confidence interval; EPO = erythropoietin; Hb = hemoglobin; TURBT = transurethral resection of a bladder tumor.

The multivariable logistic regression analysis highlighted several factors influencing the need for postoperative blood transfusions (Table 4). A higher tumor stage of the cystectomy specimen (≥pT2) was associated with a higher probability of receiving at least one postoperative blood transfusion (odds ratio [OR]: 1.37, 95% confidence interval [CI]: 1.04–1.82, *p* = 0.049). Patients with higher Hb levels at TURBT (OR: 0.99, 95% CI: 0.98–0.99, *p* = 0.017) and before cystectomy (OR: 0.98, 95% CI: 0.97–0.99, *p* < 0.001) needed fewer postoperative blood transfusions. Further, administration of neoadjuvant therapy was associated with fewer postoperative transfusions (OR: 0.70, 95% CI: 0.48–0.99, *p* = 0.05). Regarding the surgery itself, a longer operation time (by increasing the number of minutes; OR: 0.99, 95% CI: 0.99–0.99, *p* = 0.029) and a robot-assisted approach (OR: 0.46, 95% CI: 0.27–0.73, *p* = 0.002) were associated with fewer postoperative blood transfusions. No association could be confirmed for Charlson comorbidity scores, age, or the use of EPO injection, and iron or vitamin supplementation.

**Table 4:** Logistic regression for predictors of at least one postoperative blood transfusion

Variable	Odds ratio	95% CI	*p* value
*Univariable regression*			
Hb value before TURBT (g/l; continuous)	0.97	0.97–0.98	**<0.001**
Hb value before cystectomy (g/l; continuous)	0.97	0.97–0.98	**<0.001**
*Multivariable regression*			
Age (continuous)	1.01	0.99–1.03	0.09
Charlson comorbidity score (reference = 0)			
1	1.35	0.84–2.19	0.21
2	0.96	0.63–1.49	0.85
3	1.31	0.85–2.06	0.23
4	0.93	0.57–1.53	0.77
5	1.15	0.66–1.98	0.63
6	0.71	0.40–1.25	0.24
Hb value before TURBT (g/l; continuous)	0.99	0.98–0.99	**0.017**
Hb value before cystectomy (g/l; continuous)	0.98	0.97–0.99	**<0.001**
Packed red blood cells after TURBT (reference = no therapy)	1.15	0.80–1-65	0.45
EPO injection (reference = no EPO)	1.12	0.24–3.84	0.87
Iron supplementation (reference = no iron)	1.32	0.64–2.64	0.44
Vitamin B12 therapy (reference = no vitamin B12)	0.44	0.13–1.32	0.16
Folate therapy (reference = no therapy)	2.28	0.78–6.50	0.13
Neoadjuvant therapy (reference = no therapy)	0.70	0.48–0.99	**0.05**
Tumor stage cystectomy specimen ≥pT2 (reference = <pT2)	1.37	1.04–1.82	**0.049**
Surgery duration (continuous), min	0.99	0.99–0.99	**0.029**
Surgery type (reference = open)			
Laparoscopic	0.57	0.25–1.14	0.13
Robot assisted	0.46	0.27–0.73	**0.002**

CI = confidence interval; EPO = erythropoietin; Hb = hemoglobin; TURBT = transurethral resection of a bladder tumor.

Unadjusted logistic regression revealed that higher Hb levels before cystectomy were associated with a significantly lower rate of 90-d mortality (OR: 0.98, 95% CI: 0.97–0.99, *p* < 0.001). After adjusting for possible confounders, we were not able to show a significant association, but an analysis revealed that a higher ECOG score (OR: 2.55, 95% CI: 1.50–4.49, *p* < 0.001), age (OR: 1.03, 95% CI: 1.00–1.06, *p* = 0.047), administration of postoperative blood transfusions (OR: 2.01, 95% CI: 1.22–3.56, *p* = 0.007), and a higher tumor stage of the cystectomy specimen (≥pT2; OR: 2.87, 95% CI: 1.53–5.88, *p* = 0.002)) were associated with a significantly higher 90-d mortality rate (Table 5).

**Table 5:** Unadjusted and adjusted logistic regression models for 90-d mortality for Hb values before cystectomy (all patients had at least 90 d of follow-up)

Variable	OR	95% CI	*p* value
*Univariable regression*			
Hb before cystectomy (g/l)	0.98	0.97–0.99	**<0.001**
*Multivariable regression*			
Hb before cystectomy (g/l)	0.99	0.98–1.00	0.12
ECOG (0 vs ≥1)	2.55	1.50–4.49	**<0.001**
Gender (men vs women)	1.16	0.65–1.99	0.60
Age	1.03	1.00–1.06	**0.047**
Smoking status (no vs yes)	0.57	0.30–1.03	0.08
Blood transfusion after cystectomy (no vs yes)	2.01	1.22–3.56	**0.007**
Blood transfusion between TURBT and cystectomy (no vs yes)	1.30	0.67–2.42	0.42
Tumor stage cystectomy specimen (≥pT2 vs <pT2)	2.87	1.53–5.88	**0.002**
Pathological nodal stage (pN0 vs pN+)	1.83	0.94–3.40	0.07
Neoadjuvant chemotherapy (no vs yes)	0.72	0.30–1.52	0.41
Adjuvant chemotherapy (no vs yes)	0.09	0.01–0.31	**0.001**

CI = confidence interval; ECOG = Eastern Cooperative Oncology Group; Hb = hemoglobin; OR = odds ratio; TURBT = transurethral resection of a bladder tumor.

**Fig. 1:**
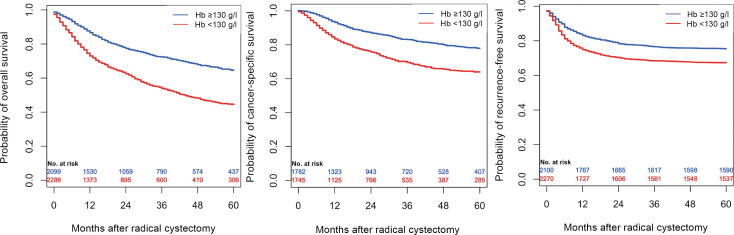
Kaplan-Meyer curves for overall, cancer-specific, and recurrence-free survival in patients with hemoglobin values >130 g/l (blue) and <130 g/l (red), and log-rank tests for overall survival (*p* < 0.001), cancer-specific survival (*p* < 0.001), and recurrence-free survival (*p* < 0.001). Hb = hemoglobin.

In an unadjusted survival analysis using the Kaplan-Meyer method, patients with preoperative Hb values <130 g/l had worse 5-yr overall survival (44% vs 65%, *p* < 0.001), worse 5-yr cancer-specific survival (64% vs 78%, *p* < 0.001), as well as 5-yr recurrence-free survival (67% vs 75%, *p* < 0.001; Fig. 1). Cox regression revealed that higher Hb values before cystectomy were associated with lower ACM (HR: 0.99, 95% CI: 0.98–0.99, *p* < 0.001), CSM (HR: 0.99, 95% CI: 0.98–0.99, *p* < 0.001), and DR (HR: 0.99, 95% CI: 0.98–0.99, *p* < 0.001; Table 6). After adjusting for predefined and previously described confounders (ECOG, gender, age, smoking status, tumor stage, and chemotherapy use), Hb values before cystectomy did not reach significant values, whereas higher Hb values before TURBT were significantly associated with lower ACM (HR: 0.99, 95% CI: 0.98–0.99, *p* = 0.002) and DR (HR: 0.99, 95% CI: 0.99–0.99, *p* = 0.025).

**Table 6:** Univariable and adjusted multivariable Cox regression models for overall mortality, cancer-specific mortality, and probability of recurrence for Hb values before cystectomy

Variable	All-cause mortality			Cancer-specific mortality			Disease relapse		
	HR	95% CI	*p* value	HR	95% CI	*p* value	HR	95% CI	*p* value
*Univariable regression*									
Hb before cystectomy (g/l)	0.99	0.98–0.99	**<0.001**	0.98	0.98–0.99	**<0.001**	0.99	0.98–0.99	**<0.001**
*Multivariable regression*									
Hb before cystectomy (g/l) Hb before TURBT (g/l)	0.99 0.99	0.99–1.00 0.98–0.99	0.94 **0.002**	0.99 0.99	0.98–1.00 0.98–1.00	0.05 0.07	0.99 0.99	0.99–1.01 0.99–0.99	0.63 **0.025**
ECOG (0 vs ≥1)	1.56	1.28–1.90	**<0.001**	1.44	1.10–1.87	**0.006**	1.18	0.96–1.45	0.11
Gender (men vs women)	1.14	0.92–1.41	0.24	1.19	0.89–1.57	0.24	1.13	0.90–1.42	0.28
Age	1.03	1.02–1.04	**<0.001**	1.01	1.00–1.03	0.08	1.02	1.01–1.03	**<0.001**
Smoking status (no vs yes)	1.14	0.92–1.41	0.22	1.25	0.95–1.65	0.11	1.16	0.92–1.45	0.20
Blood transfusion after cystectomy (no vs yes)	1.29	1.01–1.63	**0.040**	1.01	0.71–1.43	0.95	0.92	0.69–1.22	0.54
Blood transfusion between TURBT and cystectomy (no vs yes)	1.10	0.83–1.45	0.51	1.13	0.71–1.49	0.88	1.14	0.84–1.53	0.41
Tumor stage cystectomy specimen (≥pT2 vs <pT2)	2.26	1.76–2.90	**<0.001**	3.18	2.16–4.67	**<0.001**	2.89	2.16–3.85	**<0.001**
Pathological nodal stage (pN0 vs pN+)	1.43	1.11–1.86	**0.006**	1.58	1.14–2.19	**0.006**	1.69	1.31–2.18	**<0.001**
Neoadjuvant chemotherapy (no vs yes)	0.72	0.54–0.98	**0.034**	0.87	0.60–1.25	0.47	0.81	0.60–1.08	0.15
Adjuvant chemotherapy (no vs yes)	1.67	1.34–2.07	**<0.001**	2.30	1.76–3.02	**<0.001**	2.75	2.21–3.41	**<0.001**

CI = confidence interval; ECOG = Eastern Cooperative Oncology Group; Hb = hemoglobin; HR = hazard ratio; TURBT = transurethral resection of a bladder tumor.

## 4 Discussion

This large-scale, multinational study provides important insights into the prevalence, risk factors, and impact of preoperative anemia in patients undergoing radical cystectomy for bladder cancer. Our findings can be summarized in three key points: (1) anemia is prevalent in approximately 50% of patients before cystectomy, (2) several risk factors for preoperative anemia were identified, and (3) preoperative anemia is significantly associated with adverse perioperative and oncological outcomes.

Our study revealed that nearly one in two patients presented with anemia before cystectomy, which aligns with previous studies reporting anemia in 10–50% of cases [2,3]. This high prevalence underscores the critical need for a routine preoperative Hb assessment. We identified several risk factors for preoperative anemia, including advanced age, administration of neoadjuvant chemotherapy, a higher tumor stage at TURBT (≥pT2), and lower Hb levels before TURBT. These findings extend our understanding beyond previous smaller studies and provide clinicians with specific factors to identify patients who should be referred early on to patient blood management services.

Our results confirm and strengthen the association between preoperative anemia and adverse perioperative and survival outcomes. This relationship has been suggested in several previous studies [2,5,10,11], but our large, multinational cohort could confirm these finding after adjusting for several important well-described confounders. We found that anemic patients had significantly worse 5-yr overall, cancer-specific, and recurrence-free survival. Moreover, our multivariable analysis demonstrated that higher preoperative Hb levels were independently associated with lower ACM, CSM, and DR, whereas postoperative transfusions were associated with only worse 90-d mortality and ACM.

The mechanisms underlying these associations are likely multifactorial. Anemia may reflect more advanced disease or poorer overall health status. Additionally, anemia can lead to tissue hypoxia, potentially compromising wound healing and immune function, but the most worrisome hypothesis is that blood transfusions impair perioperative and oncological outcomes by transfusion-induced immunomodulation/immunosuppression with a reduction of natural killer cells and subsequent increase of tumor growth and postoperative bacterial infections [12].

Overall, anemia is often a treatable comorbidity, and despite the high prevalence and its association with poor outcomes, our study reveals a concerning underutilization of patient blood management protocols. Of the anemic patients, <25% received any form of Hb optimization, with only 4% receiving iron supplementation. This represents a significant missed opportunity, as an estimated 30% of cancer patients have iron deficiency anemia [13].

Evidence suggests that preoperative Hb optimization can improve outcomes. Intravenous iron supplementation has been shown to reduce postoperative blood transfusions and hospital stay in major abdominal surgery [14]. Even short-term protocols combining intravenous iron, EPO, vitamin B12, and folic acid supplementation have demonstrated efficacy in reducing transfusion requirements [15]. Our findings strongly support the implementation of such protocols in the perioperative management of patients undergoing radical cystectomy. However, since interventions such as EPO and vitamin B12 supplementation were administered predominantly to patients with the most severe anemia and were associated with lower preoperative Hb levels in our analysis, these findings may be subject to a selection bias. Further research is needed to determine whether these interventions are causally linked to worse outcomes or this association merely reflects the severity of anemia in patients receiving these interventions. In addition to preoperative interventions, there is conflicting evidence whether intraoperative strategies such as administration of tranexamic acid may decrease blood loss or transfusions as well as oncological outcomes [16,17].

Our analysis showed that a robotic-assisted approach was associated with fewer postoperative blood transfusions, consistent with the previous findings of randomized trials [18]. This observation leads us to hypothesize that the benefits of robotic surgery may be particularly pronounced in anemic patients. Conversely, it suggests that the advantages of robotic surgery might diminish if patients receive adequate supplementation to correct anemia preoperatively. However, due to the limited number of robotic procedures in our cohort, definitive conclusions on this matter cannot be drawn. Consequently, the selection of surgical approach should be weighed carefully against various factors, including surgeon expertise and individual patient characteristics.

This study has several limitations that warrant consideration. First, its retrospective nature introduces the potential for a selection bias and unmeasured confounding, for example, perioperative complications that could have altered postoperative outcomes were not assessed in this study. Second, the long study period (1990–2021) encompasses significant changes in surgical techniques and perioperative care, which may influence outcomes. For example, the proportion of patients who have received neoadjuvant therapy is relatively low at 18%. The reason for this is that neoadjuvant therapy was first officially recommended in the early 2000s in the USA, and in the German S3 guidelines and the European guidelines in mid-2010s.

Third, despite our large sample size, there was variability in practice patterns across centers and regions, which could affect the generalizability of our findings. Furthermore, we lacked detailed information on the specific causes of anemia in our cohort, which could provide additional insights into management strategies. Further, while our study highlights a significant association between preoperative anemia and perioperative and oncological outcomes, it does not directly assess whether anemia correction before surgery leads to improved survival. Future prospective studies are needed to clarify this relationship. Lastly, while we adjusted for several known confounders, residual confounding cannot be ruled out.

Our findings highlight several areas for future research. Prospective studies are needed to evaluate the impact of standardized preoperative anemia management protocols on outcomes following radical cystectomy. Additionally, investigation into the molecular mechanisms linking anemia to poorer oncological outcomes could reveal new therapeutic targets. Finally, cost-effectiveness analyses of different anemia management strategies would be valuable in guiding clinical practice.

## 5 Conclusions

This large, multinational study demonstrates that preoperative anemia is both common and significantly associated with adverse outcomes in patients undergoing radical cystectomy for bladder cancer. Our findings support the routine assessment of preoperative Hb levels, specifically in patients at risk of anemia. The impact of addressing anemia before surgery on perioperative and long-term oncological outcomes for patients with bladder cancer needs to be evaluated in further prospective studies.

  ***Author contributions*:** Christian Daniel Fankhauser had full access to all the data in the study and takes responsibility for the integrity of the data and the accuracy of the data analysis.

  *Study concept and design*: Fankhauser, Antonelli, Kaufmann.

*Acquisition of data*: Kaufmann, Antonelli, Afferi, Asero, Prata, Rebuffo, Veccia, Culpan, Tully, Ribeiro, Roumeguere, Hendricksen, Lambertini, Pichler, Pavan, Teoh, Roumiguié, Schulz, Soria, AlGhamlas, Desprez, Orecchia, Poyet, Alrumayyan, Rink, Zamboni, Montes, Okoye, Lo Re, Krajewski, Lavallee, Moschini.

*Analysis and interpretation of data*: Kaufmann, Fankhauser, Wettstein.

*Drafting of the manuscript*: Kaufmann, Fankhauser.

*Critical revision of the manuscript for important intellectual content*: Kaufmann, Antonelli, Afferi, Asero, Prata, Rebuffo, Veccia, Culpan, Tully, Ribeiro, Roumeguere, Hendricksen, Lambertini, Pichler, Pavan, Teoh, Roumiguié, Schulz, Soria, AlGhamlas, Desprez, Orecchia, Poyet, Alrumayyan, Rink, Zamboni, Montes, Okoye, Lo Re, Krajewski, Lavallee, Moschini, Fankhauser, Wettstein.

*Statistical analysis*: Kaufmann, Fankhauser, Wettstein.

*Obtaining funding*: None.

*Administrative, technical, or material support*: Fankhauser, Antonelli.

*Supervision*: Fankhauser.

*Other*: None.

  ***Financial disclosures:*** Christian Daniel Fankhauser certifies that all conflicts of interest, including specific financial interests and relationships and affiliations relevant to the subject matter or materials discussed in the manuscript (eg, employment/affiliation, grants or funding, consultancies, honoraria, stock ownership or options, expert testimony, royalties, or patents filed, received, or pending), are the following: None.

  ***Funding/Support and role of the sponsor*:** None.
