# Supplementary material for: Prevalence and Prognostic Significance of Preoperative Anemia in Radical Cystectomy Patients: A Multicenter Retrospective Observational Study
Source: Eur Urol Open Sci. 2025 Jun 4;77:10–8. doi: 10.1016/j.euros.2025.05.002 (PMC12169704; doi:10.1016/j.euros.2025.05.002)
Supplement: Supplementary Data 1 [file mmc1.docx]

**Supplementary material**

Key clinical definitions included: ECOG performance status [1] and ASA score [2] to define clinical performance. Comorbidities were quantified using Charlson Comorbidity score [3]. The 1973 [4] and 2004 [5] WHO grading scheme, as well as on the TNM staging 2017 defined by the American Joint Committee on Cancer [6] were adopted for cancer staging. Renal disease was defined as a serum creatinine level of above 200 *µ*mol/l or 2.26 mg/dl, dialysis requirement or renal transplant. Conversely, liver disease diagnosis was based on the presence of total bilirubin levels elevated two-fold above normality in combination with elevated serum alanine aminotransferase (ALT), aspartate aminotransferase (AST) or alkaline phosphatase (AP) levels three-times above the cut-off of normality or the presence of cirrhosis. Alcohol abuse was defined as alcoholic beverage consumption of more than eight drinks a week. Bleeding was classified according to the European Society of Cardiology [7].

**Variables included for statistical analysis**

The following variables were included for statistical analysis after previous drafting of directed acyclic graph (DAG) (Supplementary Fig. 1) and a literature search and missing data was handled as follows:: Age (continuous), exclusion if missing data, ASA score (categorial, 0-4), exclusion if missing data, Charlson Comorbidity score (categorial, 0-6) exclusion, if missing data, ECOG performance status was grouped in 2 groups (0 vs. >=1), exclusion if missing data, tumor stage at TURBT and at the cystectomy specimen was grouped in 2 groups (<pT2, >/=pT2), exclusion, if missing data, clinical and pathological nodal stage (c/pN0 versus c/pN+) if missing, cN0 assumed, exclusion if no pathological modal stage, Hb at TURBT (continuous), exclusion, if missing data, Hb before cystectomy (continuous), exclusion, if missing data, EPO injections (0=no, 1=yes), if missing data, no EPO (0) was assumed, oral or i.v iron supplementation (0=no, 1=yes), if missing data, no therapy (0) was assumed, Vitamin B12 therapy (0=no, 1=yes), if missing data, no therapy (0) was assumed, Folate therapy (0=no, 1=yes), if missing data, no therapy (0) was assumed, Alcohol abuse (> 8 drinks/week) 0=no, 1=yes, ), if missing data, no abuse (0) was assumed, Diabetes (0=no, 1=yes), if missing data, no disease (0) was assumed, smoking history (0=non-smoker, 1=current, 2=ex-smoker), if missing data, non-smoker (0) was assumed, (Neo)adjuvant treatment (0=no, 1=yes), if missing data, no therapy (0) was assumed. Surgery duration (continuous), exclusion, if missing data, surgery type (categorial, open, laparoscopic, robot-assisted), exclusion, if missing data.

**Supplementary Table 1: Possible Confounders for Cox Regression**

|  |  | **Significant influence on oncological outcomes** | | |  |
| --- | --- | --- | --- | --- | --- |
| **Paper** | **No. Pat.** | **OS** | **CSS** | **RFS** | **Pred. periop transf** |
| Jo 2016 Int Urol Nephrol , DOI: 10.1007/s11255-016-1219-x  **🡪 Preop anemia** | 200 | Age, ECOG >1, preop. Hb | ECOG >1 | ECOG >1, preop Hb | - |
| Gierth 2015 J Cancer Res Clin Oncol DOI: 10.1007/s00432-015-1957-7  **🡪 preop anemia** | 684 | Age, ECOG >1, pathologic stage (advanced), pN+, positive surg margin, preop Hb, transfusion of EC | Age, gender, ECOG >1, pathologic stage (advanced), pN+, positive surg margin, preop Hb | Age, gender, ECOG >1, pathologic stage (advanced), pN+, positive surg margin, preop Hb | - |
| Ferran-Carpintero Actas Urol Esp 2020, DOI: 10.1016/j.acuro.2020.04.005  🡪 **preop** **anemia** | 176 | **Univar**: ASA 4, preop Anemia, preop Ektasia, >pT2, pN+  **Multivar**: preop Anemia, >pT2, pN+ | - | - | - |
| Syan-Bhanvadia, Urologic Oncology: Seminars and Original Investigations 2017  🡪 **periop blood transfusions** | 173 | **Multivar**: Transfusion, >=pT3, pN+, adjuvant chemo, age, preop hemoglobin | - | **Multivar**: Transfusion, >=pT3, pN+, adjuvant chemo, age, | **Multivar:** Age, CCI >2, neoadjuvant therapy, >=pT3, est. blood loss |
| Sui, International Journal of Urology 2016  **🡪 periop blood transfusions** | 2934 | **Outcome Mortality: Multivar:** Age, BMI>40, Smoking | - | **-** | **-** |
| Linder European urology 2013  **🡪 periop blood transfusions** | 2060 | Age, Gender (male), ECOG, preop Hb, periop EC transfusion, >=pT2, pN+, | ECOG, preop Hb, periop EC transfusion, >=pT2, pN+, | >=pT2, pN+, |  |
| Kluth, BJU international 2014  **🡪 periop blood transfusions** | 2895 | **Multivar**: >=pT2, pN+, soft tissue surgical margin, LVI, adjuvant chemotherapy (positive) | Age, >=pT2, pN+, soft tissue surgical margin, LVI, adjuvant chemotherapy (positive) | Age, >=pT2, pN+, soft tissue surgical margin, LVI, adjuvant chemotherapy (positive) | **-** |

**Supplementary Figure 1 Histogramm and density plot of Hb values before TURBT and radical cystectomy**

**
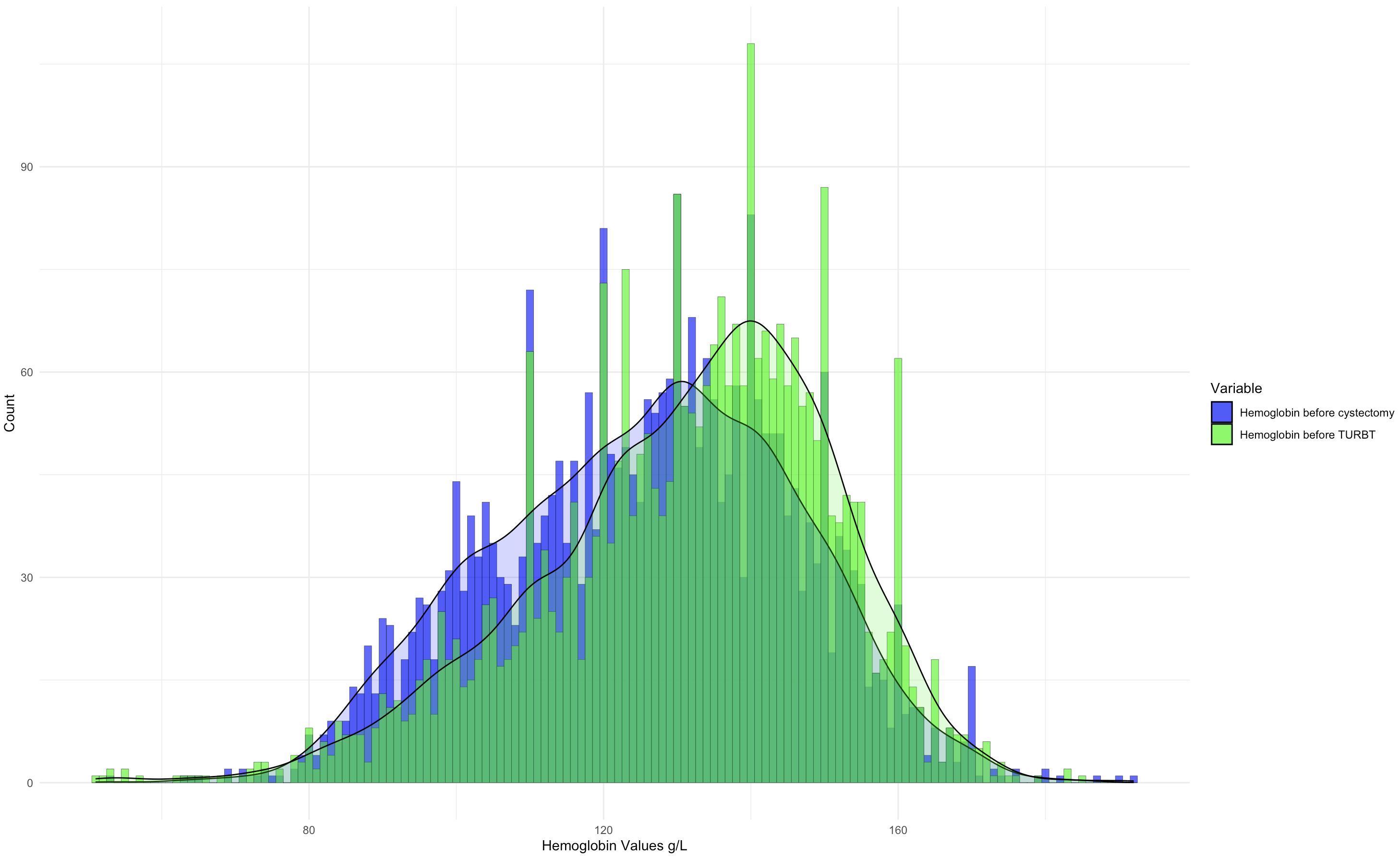
**

**Supplementary Figure 2 Directed acyclic graph for Table 3: Linear Regression Predictors for hemoglobin before cystectomy**


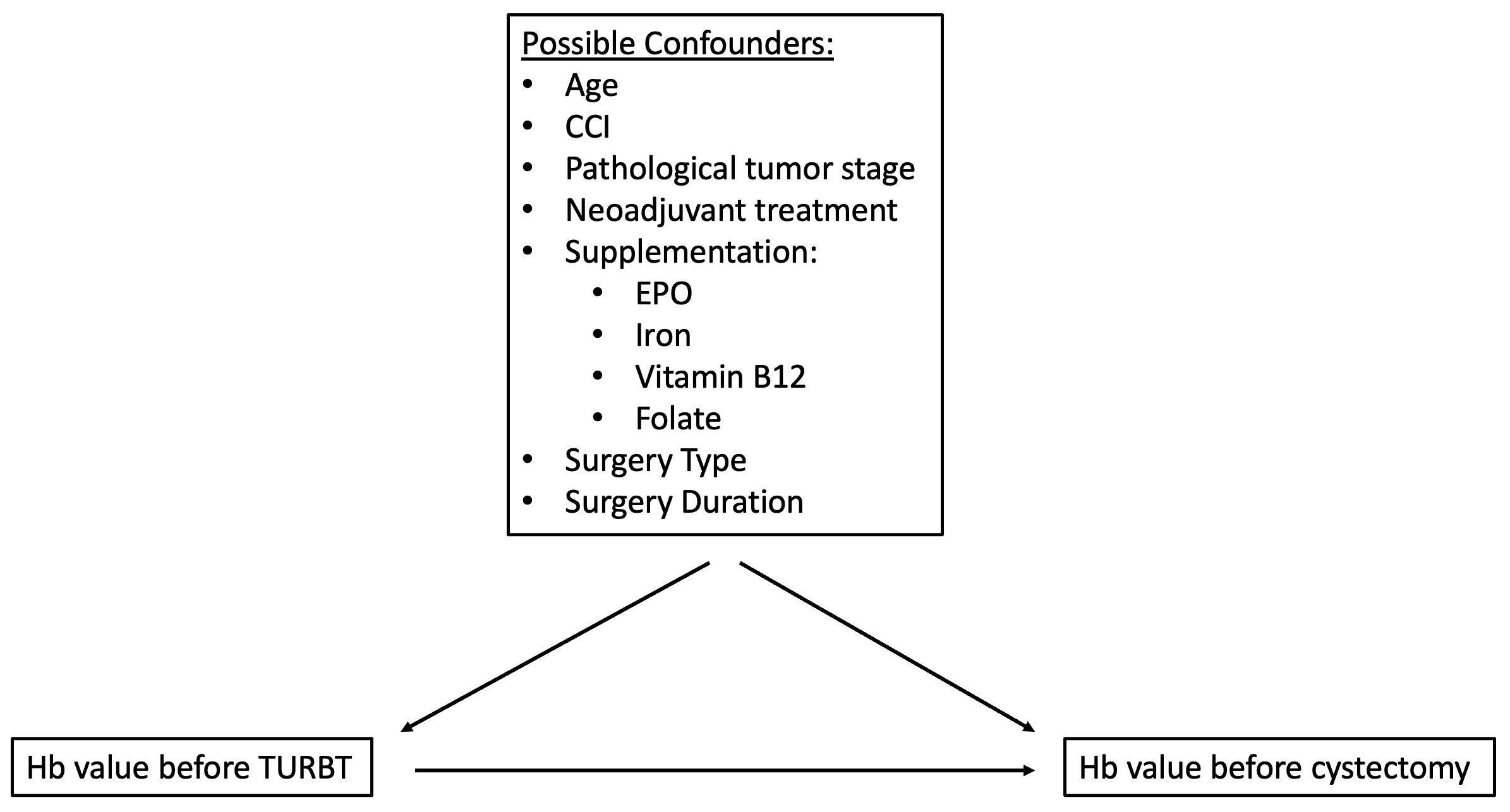


**Supplementary Figure 3** **Directed acyclic graph for Table 4: Cox Regression for predictors of at least 1 postop. Transfusions**


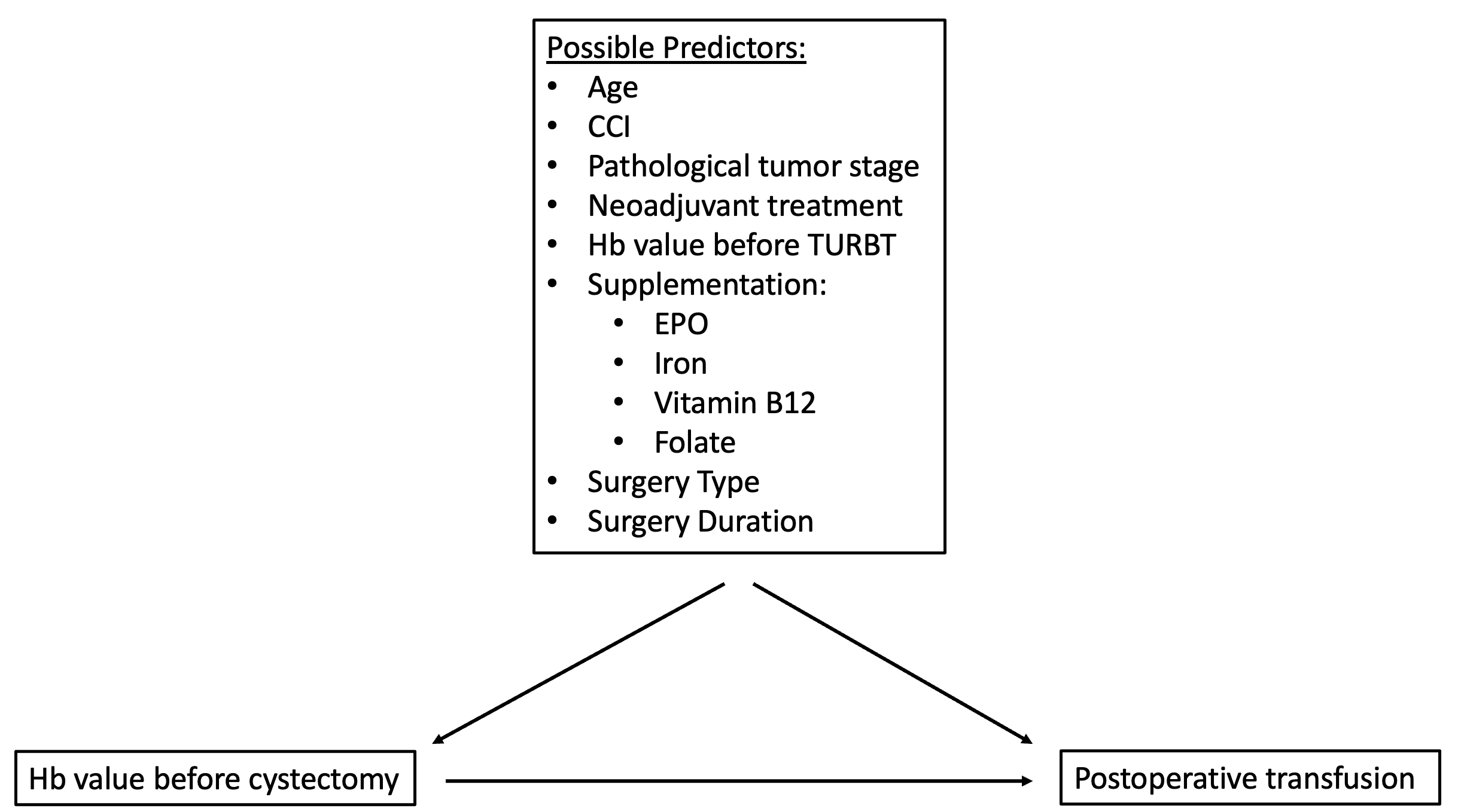


**Supplementary Figure 4** **Directed acyclic graph for Table 5/6: Cox Regression „Is hemoglobin before cystectomy a predictor of survival endpoints“**


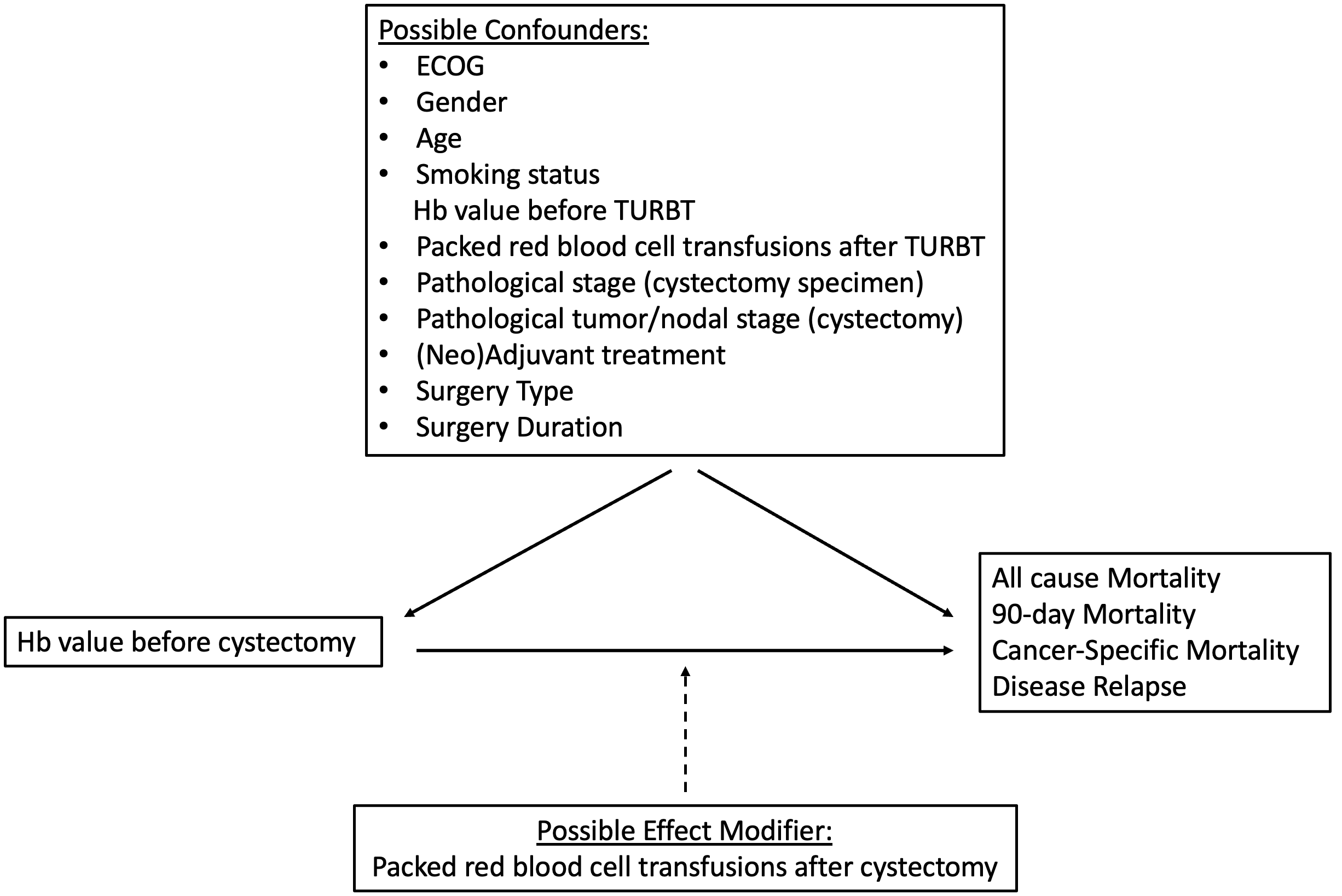


**^**

[1] Buccheri G, Ferrigno D, Tamburini M. Karnofsky and ECOG performance status scoring in lung cancer: a prospective, longitudinal study of 536 patients from a single institution. *Eur J Cancer*. Jun 1996;32A(7):1135-41. doi:10.1016/0959-8049(95)00664-8

[2] Saklad M. GRADING OF PATIENTS FOR SURGICAL PROCEDURES. *Anesthesiology*. 1941:281-284.

[3] Charlson ME, Pompei P, Ales KL, MacKenzie CR. A new method of classifying prognostic comorbidity in longitudinal studies: development and validation. *J Chronic Dis*. 1987;40(5):373-83. doi:10.1016/0021-9681(87)90171-8

[4] Mostofi FK, Sobin, Leslie H, Torloni, Humberto & World Health Organization. (‎1973)‎. Histological typing of urinary bladder tumours / F. K. Mostofi, in collaboration with L. H. Sobin, H. Torloni and pathologists in fourteen countries. World Health

[5] Epstein JI, Amin MB, Reuter VR, Mostofi FK. The World Health Organization/International Society of Urological Pathology consensus classification of urothelial (transitional cell) neoplasms of the urinary bladder. Bladder Consensus Conference Committee. *Am J Surg Pathol*. Dec 1998;22(12):1435-48. doi:10.1097/00000478-199812000-00001

[6] Amin MB, Greene FL, Edge SB, et al. The Eighth Edition AJCC Cancer Staging Manual: Continuing to build a bridge from a population-based to a more "personalized" approach to cancer staging. *CA Cancer J Clin*. Mar 2017;67(2):93-99. doi:10.3322/caac.21388

[7] Mehran R, Rao SV, Bhatt DL, et al. Standardized bleeding definitions for cardiovascular clinical trials: a consensus report from the Bleeding Academic Research Consortium. *Circulation*. Jun 14 2011;123(23):2736-47. doi:10.1161/CIRCULATIONAHA.110.009449
